# A Rare Case of Eustachian Valve Endocarditis in a Young Male With Poorly Controlled Type 1 Diabetes Mellitus

**DOI:** 10.7759/cureus.25314

**Published:** 2022-05-25

**Authors:** Hardik Fichadiya, Kalpesh K Shah, Muhammad Atif Masood Noori, Harshwardhan Khandait, Payal Rath, Asnia Latif, Ruchi Patel, Raja Pullatt

**Affiliations:** 1 Internal Medicine, Rutgers University New Jersey Medical School, Elizabeth, USA; 2 Cardiology, St. Joseph Medical Center, Paterson, USA; 3 Medicine, St. George's University School of Medicine, St. George's, GRD; 4 Cardiology/Internal Medicine, Rutgers University New Jersey Medical School, Elizabeth, USA

**Keywords:** cefazolin, transesophageal echo, mssa bacteremia, diabetes mellitus, eustachian valve endocarditis

## Abstract

The eustachian valve (EV) is a vestigial structure found at the junction of the inferior vena cava and the right atrium, a remnant of the embryological sinus venosus that may persist throughout life. Right-sided infective endocarditis of the eustachian valve remains a distinctly rare and under-diagnosed entity.

Commonly known risk factors of eustachian valve endocarditis (EVE) are intravenous drug use, in-dwelling intracardiac devices, and central lines, although more recently immunocompromised states, e.g. uncontrolled diabetes mellitus and old age, have been recognized as risk factors for the disease. Although *Staphylococcus aureus* has been the most commonly implicated organism, cases of infections with gram-negative organisms are emerging.

We present a 47-year-old male with uncontrolled type 1 DM who initially presented to the ED with complaints of low back pain and dysuria and was later found to have eustachian valve endocarditis ultimately treated with intravenous antibiotics.

## Introduction

The eustachian valve (EV) is a vestigial structure found at the junction of the inferior vena cava and the right atrium. EV directs oxygenated blood through the foramen ovale into the left atrium in embryonic life. Often present only as a rudimentary ridge, EV can vary in thickness and persist into adult life. EV endocarditis (EVE), rarely diagnosed, is most commonly found in intravenous drug users. Other risk factors include in-dwelling right-sided cardiac hardware and central lines [1]. EV endocarditis was first described by Edwards et al. in 1986, and about 30 cases have been reported so far [2]. Here, we describe a case of EVE in a young male with uncontrolled diabetes. 

## Case presentation

A 47-year-old male with a past medical history of poorly controlled type 1 diabetes mellitus presented to the emergency department with lower back pain and dysuria. On presentation, he was hypotensive (BP: 90/60 mmHg), tachycardic (HR: 130s), tachypneic (RR: 24/min) with a temperature of 95.6 F. On physical exam, the patient had dry mucous membranes and a distended urinary bladder up to the umbilicus. Labs revealed a total white blood cell count of 29,000/mm3 with 82% polymorphs, serum glucose of 973 mg/dl, bicarbonate of 4 mEq/L, and arterial pH of 6.9; lactic acid was 2.1 mg/dl. Urinalysis after insertion of a Foley catheter showed an abundance of WBCs per high-power field, moderate bacteria, small leukocyte esterase, and negative nitrites. 

Code sepsis was called, and two sets of blood cultures were drawn 15 minutes apart. Intravenous fluids, insulin, and antibiotics, including vancomycin and cefepime, were started. 

Given the underlying renal insufficiency, a non-contrast CT scan of the spine was ordered and showed degenerative disc disease, but no evidence of infection. CT abdomen without contrast showed signs of chronic bladder outlet obstruction with a distended wall and moderate bilateral hydronephrosis. 

**Figure 1:**
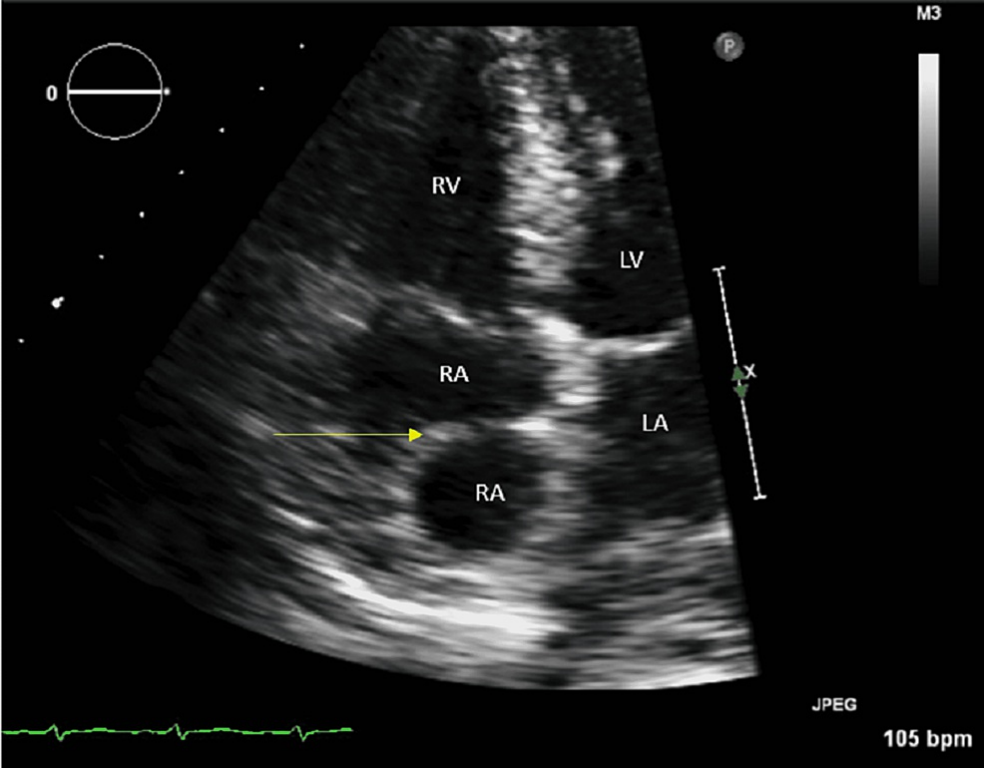
Transthoracic echocardiogram (TTE) ECHO showing a mass in the right atrium (yellow arrow) LA, left atrium; LV, left ventricle; RA, right atrium; RV, right ventricle

A transthoracic echocardiogram (TTE) showed a fragile, mobile mass in the right atrium. It was unclear if the mass was a thrombus, vegetation, or a part of an aneurysm from the inter-atrial septum (Figure 1).

**Figure 2:**
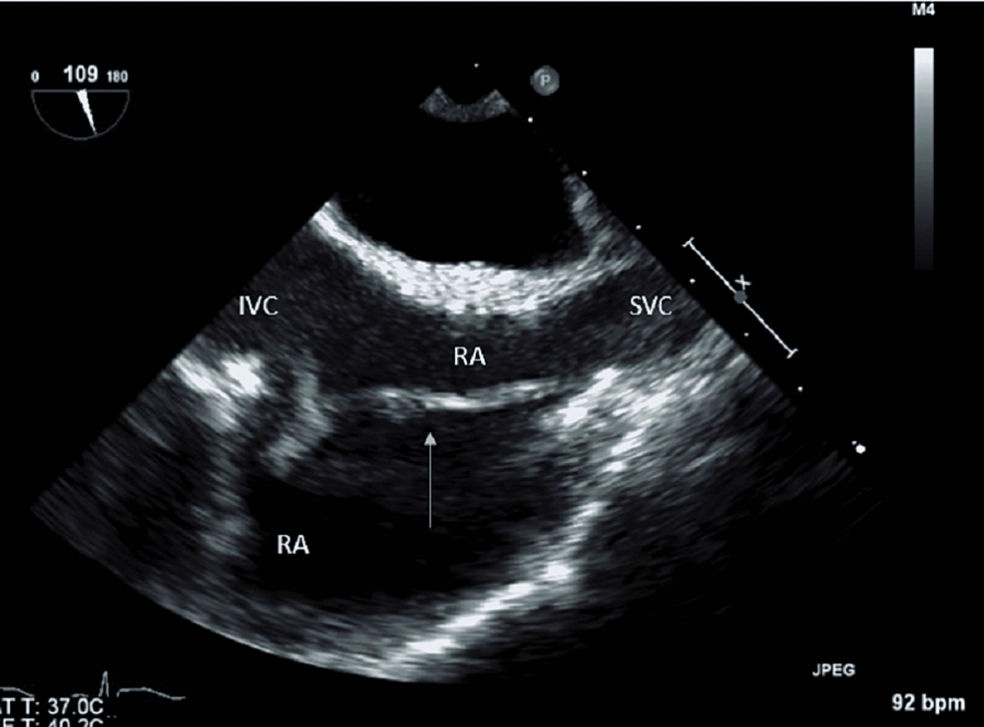
Initial trans-esophageal echocardiogram (TEE) showing the biofilm extending along the right atrium and associated with a peduncle. RA, right atrium; IVC, inferior vena cava; SVC, superior vena cava

**Figure 3:**
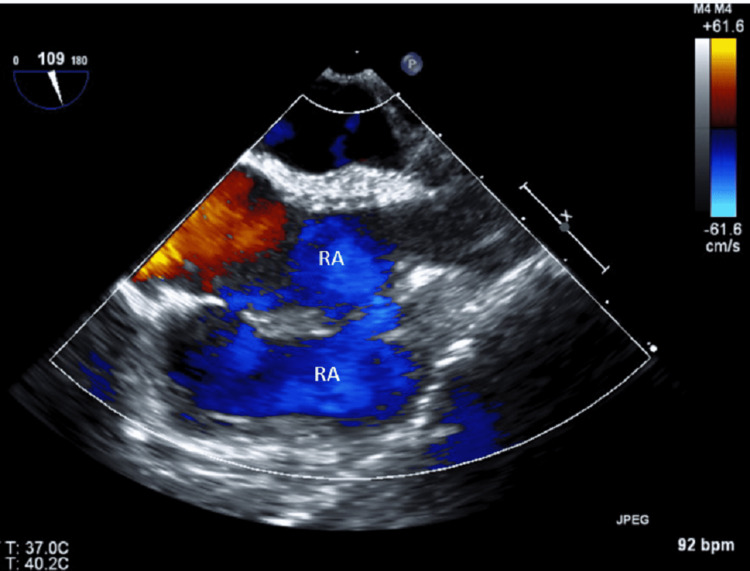
Trans-esophageal echocardiogram (TEE) Doppler film indicating the right atrium as a single cavity on both sides. RA, right atrium

Subsequently, a trans-esophageal echocardiogram (TEE) was performed, revealing a 1.5 cm linear, mobile vegetation on the eustachian valve with a biofilm extending to the superior vena cava (Figures 2, 3). 

Blood culture results showed methicillin-sensitive *Staphylococcus aureus* (MSSA) sensitive to oxacillin. Urine cultures had grown 15,000 colony-forming units of MSSA. The preferred antibiotic was intravenous cefazolin 2gm every 8 hours. 

Cardiology was on board, and a trans-esophageal echocardiogram to monitor the vegetation was planned in one week. 

Although the patient was receiving the antibiotics, he continued to be febrile and tachycardic. He developed a left flank and lower quadrant abdominal pain three days later. CT scan showed a newly developed abscess-fluid collection around the left hip extending from the left obturator internist muscle to the acetabulum and around the ischial tuberosity, extending between left gluteus minimus and maximus muscles. No joint effusion was noted. Abscesses were noted in both kidneys, likely secondary to septic emboli from the endocarditis. The drained abscesses grew MSSA on culture. 

**Figure 4:**
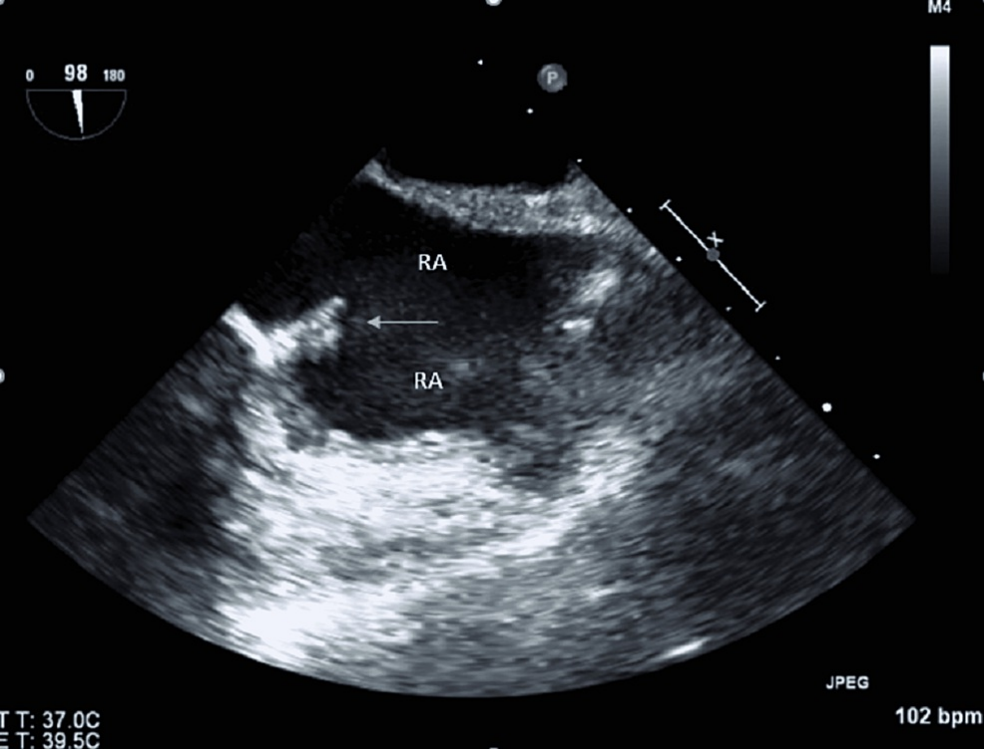
Trans-esophageal echocardiogram (TEE) showing a decrease in the size of the biofilm in the right atrium after the antibiotic therapy. RA, right atrium

After drainage, the patient reported improvement in pain and his tachycardia was resolving. A repeat trans-esophageal echocardiogram performed 10 days after antibiotics initiation showed a decrease in the size of the right atrial mass (Figure 4). 

A peripherally inserted central line was placed, and the patient was discharged on six weeks of intravenous cefazolin to sub-acute rehab. A repeat trans-thoracic echocardiogram did not show any evidence of endocarditis six weeks later. 

## Discussion

Infective endocarditis (IE) is defined as an infection of the endocardial surfaces of the heart (mainly the heart valves native/prosthetic or the mural endocardium). The incidence of IE ranges from 3-10 per 100,000 per year, with studies indicating increasing incidence in recent years [3]. 

Historically, patients with rheumatic heart disease (RHD) or adults with congenital heart disease have been at the most significant risk [4]. However, with the use of newer antibiotics and a decline in RHD incidence, there has been a change in trend, with older patients being more commonly affected and Staphylococcus surpassing oral Streptococci to be the most common cause of the disease [4]. Risk factors include prosthetic heart valves, hemodialysis catheters, venous catheters, and intravenous drug abuse [1]. 

The eustachian valve is a remnant of the sinus venosus, which in fetal life shunts blood from the inferior vena cava (IVC) to the left atrium via the foramen ovale. It is located at the junction of the right atrium and the IVC; however, in adults, it is non-functional. Right-sided IE is much less common than the left [5]. Eustachian valve endocarditis (EVE) falls under right-sided IE.

It is a rare entity, first described by Edwards et al. in 1986, with only 29 cases reported henceforth [2]. The exact incidence of eustachian valve IE is unknown, but a retrospective review by Roman et al. in 2001 reported an incidence of 3.3% in right-sided IE [3]. The most common risk factor for eustachian valve endocarditis is intravenous drug use, whereas central venous catheters, pacemakers, congenital heart disease, history of rheumatic heart disease, chronic alcoholism, and HIV are also associated with increased risk [5].

Typically, EVE occurs in isolation [6], but cases with simultaneous involvement of tricuspid and mitral valves have been reported [7]. 

Endothelial injury is the trigger for IE. Subsequently, there is a release of inflammatory mediators such as cytokines, tissue factors with the formation of platelet-fibrin thrombus, and bacterial adherence. Bacterial colonization triggers further endothelial injury, eventually leading to infected vegetation. *Staphylococcus aureus* has been the most commonly implicated organism in EVE patients [7]; cases involving other organisms such as *Staphylococcus hominis*, *Enterococcus cloacae*, *E. coli* [8], *Streptococcus viridans* [9], *Klebsiella pneumonia* [10,11], and *Actinomyces israelii* [12] have also been reported. 

Clinical presentation is similar to right-sided endocarditis, including fever, shortness of breath, chest pain, and cough. The pulmonary manifestations result from septic emboli to the lungs. Patients can also present with signs of right heart failure, including jugular venous distension and pedal edema. EVE should be suspected in patients presenting with septic pulmonary emboli in the absence of vegetations on the tricuspid valve. 

Although TTE is the preferred initial investigation, TEE has higher sensitivity for detecting eustachian valve lesions [7,11] and hence should be performed on all patients with high clinical suspicion and unremarkable TTE. TEE, in addition, also helps differentiate eustachian valve lesions from vegetations, thrombus, and masses arising from the interatrial septum. 

Currently, there are no specific established guidelines for managing eustachian valve endocarditis, and treatment is similar to right-sided endocarditis. As per the European Society of Cardiology, intravenous antibiotics and diuretics are the cornerstones of therapy [13]. The choice of empiric antibiotics is based on patient characteristics and type of intravenous drug use [13]. Antibiotics are then tailored according to blood culture and sensitivity results.

For uncomplicated IE or when MSSA is suspected, nafcillin, oxacillin, or cefazolin are preferred over vancomycin or daptomycin [14]. Vancomycin should be used for complicated IE or when methicillin-resistant *Staphylococcus aureus* is suspected. Surgery is indicated in cases where IE is caused by organisms difficult to eradicate, patients are unresponsive to medical therapy, and the vegetation exceeds 20mm in size [15]. 

As with tricuspid valve endocarditis, pulmonary embolism is a known compilation of EVE.

Our case represents isolated eustachian valve endocarditis from methicillin-sensitive *Staphylococcus aureus*. The endocarditis was likely secondary to the urinary tract infection (UTI) as the patient had no traditional risk factors for right-sided IE (IV drug use or central venous catheter). To the best of our knowledge, this is the first case of eustachian valve endocarditis secondary to a UTI.

## Conclusions

Eustachian valve endocarditis is a well-documented phenomenon that remains extremely rare. The low incidence, in part, is explained by under-diagnosis and under-reporting. Transthoracic echo is rarely diagnostic. In all right-sided endocarditis cases with the equivocal location of vegetation, a transesophageal echo must be performed to look for EVE. A negative TTE should not be used to rule out the diagnosis. 

In addition to the conventional risk factors like intravenous drug use and hardware, immunocompromised states, such as old age and uncontrolled diabetes, should also be considered while evaluating the risk factors for this diagnosis. 
